# Exploring power and parameter estimation of the BiSSE method for analyzing species diversification

**DOI:** 10.1186/1471-2148-13-38

**Published:** 2013-02-11

**Authors:** Matthew P Davis, Peter E Midford, Wayne Maddison

**Affiliations:** 1 The Field Museum, 1400 South Lake Shore Drive, Chicago, IL, 60605, USA; 2 NESCent: National Evolutionary Synthesis Center, 2024 W. Main Street, Suite A200, Durham, NC, 27705-4667, USA; 3 University of British Columbia, 4200-6270 University Blvd, Vancouver, B.C, Canada, V6T 1Z4

**Keywords:** Key innovations, Character evolution, Systematics

## Abstract

**Background:**

There has been a considerable increase in studies investigating rates of diversification and character evolution, with one of the promising techniques being the BiSSE method (binary state speciation and extinction). This study uses simulations under a variety of different sample sizes (number of tips) and asymmetries of rate (speciation, extinction, character change) to determine BiSSE’s ability to test hypotheses, and investigate whether the method is susceptible to confounding effects.

**Results:**

We found that the power of the BiSSE method is severely affected by both sample size and high tip ratio bias (one character state dominates among observed tips). Sample size and high tip ratio bias also reduced accuracy and precision of parameter estimation, and resulted in the inability to infer which rate asymmetry caused the excess of a character state. In low tip ratio bias scenarios with appropriate tip sample size, BiSSE accurately estimated the rate asymmetry causing character state excess, avoiding the issue of confounding effects.

**Conclusions:**

Based on our findings, we recommend that future studies utilizing BiSSE that have fewer than 300 terminals and/or have datasets where high tip ratio bias is observed (i.e., fewer than 10% of species are of one character state) should be extremely cautious with the interpretation of hypothesis testing results.

## Background

Maddison et al. [1] described a method using a binary-state speciation and extinction model (BiSSE) that estimates rates of change in a binary character and rates of speciation and extinction contingent on the character state, given a known distribution of observed states on the tips of a tree of contemporaneous species. The BiSSE method assumes that the tree includes all extant species and that all species have known data for the state of the single binary character [1]. FitzJohn et al. [2] provided additional methodology for including known species diversity into an incomplete phylogeny if a researcher could confidently place taxa into unresolved terminal clades. BiSSE provides estimates for the rates of speciation in each character state (λ_0_, λ_1_), extinction in each state (μ_0_, μ_1_), and character transition rates between states (*q*_01_, *q*_10_). Such estimates are important to studies of whether a particular feature is controlling the diversification rates of clades and whether the effect is on speciation, extinction, or both [3-5]. Recent studies have also modified the basic approach of the BiSSE method to estimate further parameters associated with quantitative characters [6] and geography [7].

Maddison et al. [1] discussed the development of the BiSSE method and demonstrated its ability to estimate rates from simulated data sets. Recently there has been a burst in the number of studies that have utilized the BiSSE method to explore rates of diversification in various taxonomic groups for the purpose of testing hypotheses involving key innovations and the evolution of characters [8-19]. However, the majority of these studies explore diversification and character evolution hypotheses with fewer than 200 taxa, despite the initial warning by Maddison et al. [1] that the power of analysis may be affected by low sample size.

Wise use of any statistical method should be guided by an understanding of its power and ability to distinguish hypotheses of interest, but current empirical studies lack sufficient guidance, because there has been little work on the BiSSE method’s behavior under different sample sizes (numbers of species) and parameter values. The primary goal of this study is to explore the power and accuracy of parameter estimation of the BiSSE method. Using simulations we explore the number of species needed to obtain good power, the advantages of estimating fewer parameters, and the effect of the extreme asymmetries in rates. Additionally, because there are many ways that an observed excess of a character state can be explained through macroevolutionary processes (e.g., increased speciation rates in taxa with State 1, higher extinction rates in taxa with State 0), there is also concern regarding confounding effects [20], and whether BiSSE can identify which rate asymmetries are causing the observed character excess in empirical data.

## Results

### Power of BiSSE method

#### Asymmetries in speciation rate

**Figure 1:**
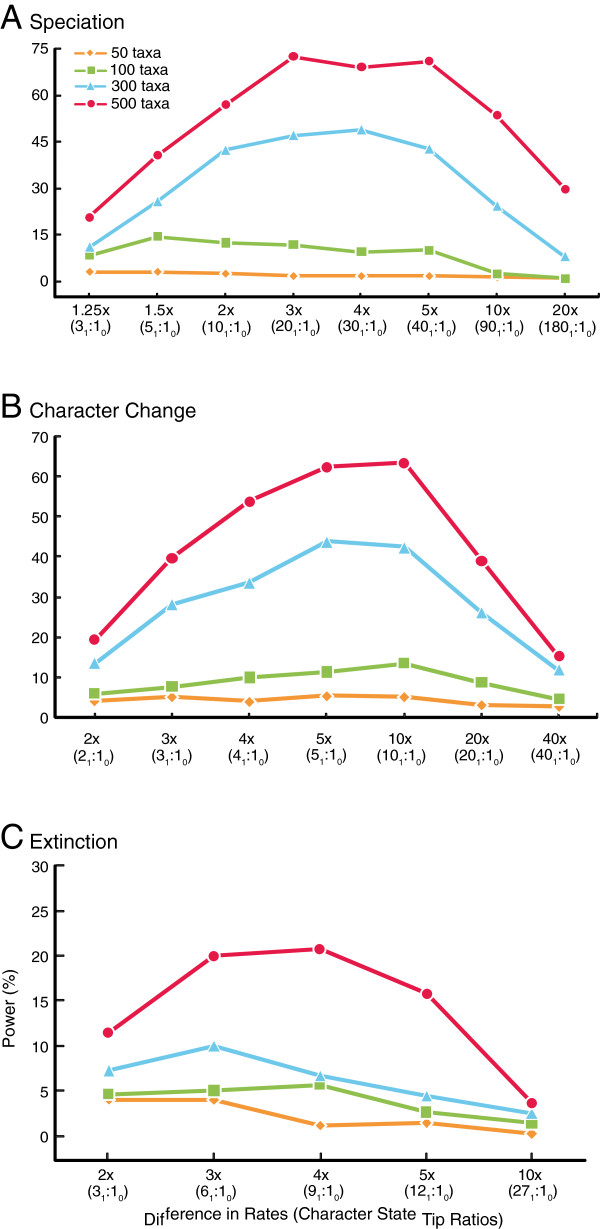
**Power of BiSSE under simulations with asymmetrical rates of (a) speciation, (b) character change, (c) extinction.** See Table 1 and Additional file 1: Table S1-S3 for list of rate values.

Extremely low power (>5%) was observed when tree size included 50 taxa, regardless of the degree of rate asymmetry (Figure 1a, Additional file 1: Table S1). Power marginally improved for tree sizes of 100 taxa, but decreased considerably as the asymmetry increased to 10 and 20× rate difference (Figure 1a). A tree size of 300 tips indicated a higher overall power for each difference in rate than those observed with 50 or 100 taxa. Power increased as the degree of difference in rate asymmetry increased, until reaching a rate difference of four times the speciation rate where the power begins to decrease as the degree of rate asymmetry grows. This same pattern was observed for a tree size of 500 tips (Figure 1a). In general, power increased as tree size increased (Figure 1a), and a pattern of power decrease following an increase in tip-ratio bias resulting from rate asymmetry was observed in the simulation including more tips (Figure 1a).

#### Asymmetries in rate of character state change

For each asymmetrical model of character change (e.g., 2×, 5×), power increased with an increase in tree size (Figure 1b). Power remained low and did not increase with a difference in rates when the tree size was 50 taxa. There was a slight increase in power as the degree of difference in rates increased with tree sizes of 100 taxa (Figure 1b). Power was higher for simulations with 300 taxa for each respective difference in rates compared to the same simulations with 100 and 50 taxa. Power increased as the rate difference increased to 5× then leveled off to 10×, followed by a strong decrease in power as the difference in rates increased to 20× and 40× (Figure 1b). This same pattern was observed in simulations of 500 taxa and in general there was a decrease in power observed in all tip sizes beyond a 10x difference in rates of character change (Figure 1b).

#### Asymmetries in extinction rate

As with rates of speciation and character state change, power increased as tree size increased regardless of the amount of difference in extinction rate (Figure 1c). With tree sizes of 50 taxa, power was low regardless of the degree of rate asymmetry, and power was similarly low with 100 taxa. With tree sizes of 300 and 500 taxa, power increased until a rate difference of 3×, with power decreasing as the degree of rate asymmetry continue to increase. Overall, power in hypothesis testing with extinction rates was lower than those for speciation or character state change (Figure 1c).

#### Power of six parameter model vs. four parameter model

**Figure 2:**
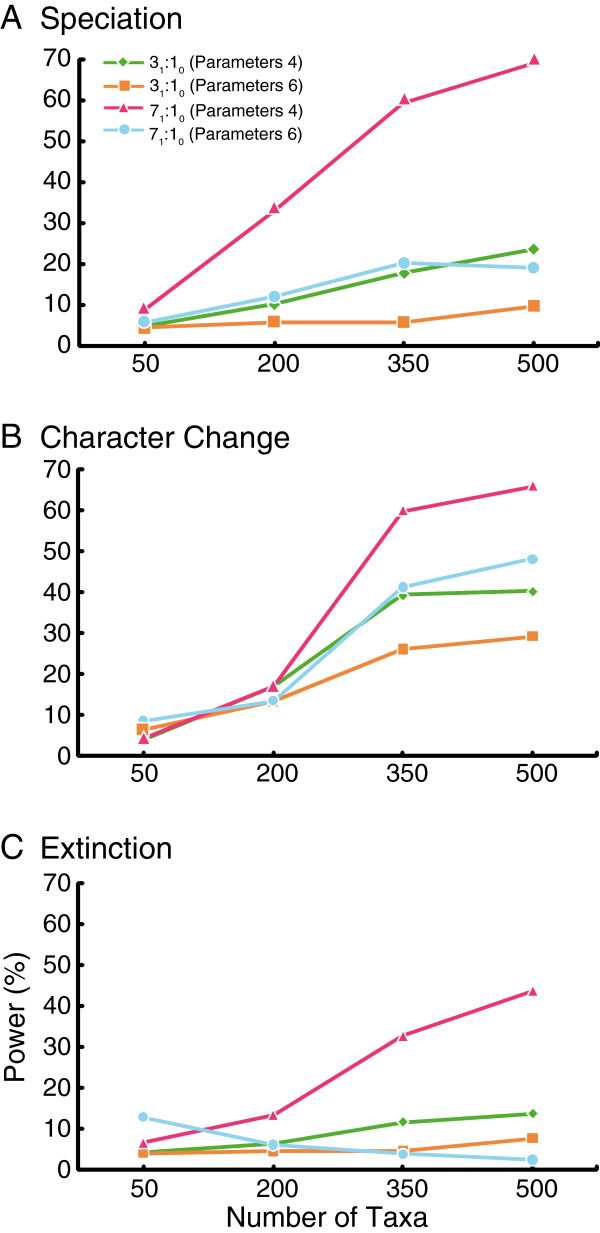
**Power of BiSSE under four versus six parameter models under low (3**_**1**_**:1**_**0**_**) and high (7**_**1**_**:1**_**0**_**) character state bias as the result of rate asymmetries in (a) speciation, (b) character change, and (c) extinction.** See Additional file 1: Table S4 for rate values.

The change in power among low and high tip bias scenarios when using a model that estimates fewer parameters is observed in Figure 2 (Additional file 1: Table S4). The reduced four parameter model differs from the full six parameter model (λ_0_, λ_1_, μ_0_, μ_1_, *q*_01_, *q*_10_) with two of the rates constrained to be equal in both character states, for example (λ_0_, λ_1_, μ_0_=μ_1_, *q*_01_=*q*_10_). In nearly all cases, especially with tree sizes greater than 300 taxa, there was an increase in power when the four parameter model was used compared to the six parameter model (Figure 2). The greatest increase in power occurred in the scenarios with a greater degree of bias (7:1 State 1 favored), and the effect was observed regardless of which parameter possessed the rate asymmetry (Figure 2, Additional file 1: Table S4). As was the case with the six parameter estimations, power is low when tree size is below 300 taxa with a four parameter model.

### Parameter estimation

#### Estimating parameters under asymmetrical speciation

**Figure 3:**
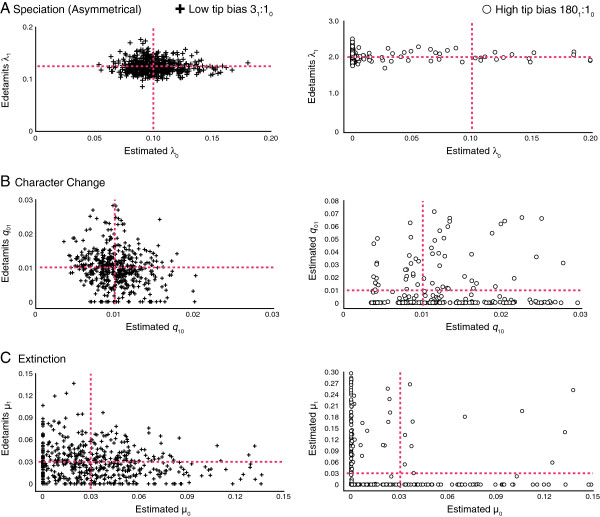
**Parameter estimations of (a) speciation, (b) character change, and (c) extinction under different degrees of asymmetry in speciation rates with corresponding tip ratios.** Point of intersection between red lines represents known values.

As previously identified by Maddison et al. (2007), the BiSSE method estimates speciation rates well, with strong delimitation of known asymmetrical rates given an appropriate tree size (Figure 3a), although precision decreases as the speciation rate asymmetry, and tip bias, increases. For estimates of known symmetrical rates of character change under asymmetrical speciation rates, accuracy and precision of estimating the known rates become significantly worse under the higher tip bias scenario (Figure 3b). A similar pattern is recovered for estimates of known symmetrical extinction values where rates were estimated with more accuracy and precision under low tip bias (asymmetrical speciation rate of 1.25), with accuracy and precision strongly decreasing under high tip bias (20× asymmetry in speciation rates) as observed in Figure 3c. Accuracy and precision of parameter estimation greatly decreases for all rates with a small sample size of tips, regardless of low or high tip bias (Additional file 2: Figure S1).

#### Estimating parameters under asymmetrical character change

**Figure 4:**
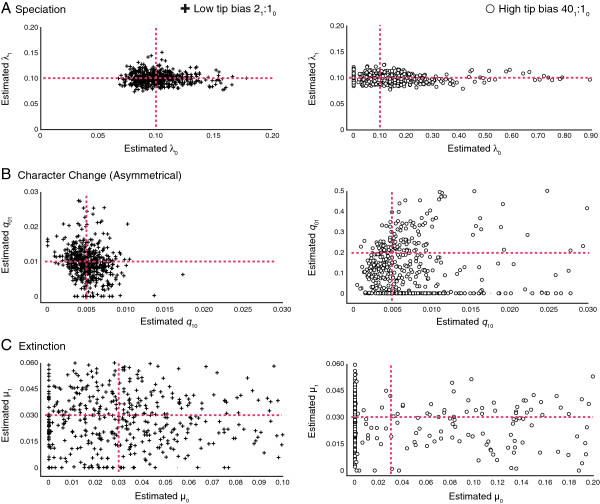
**Parameter estimations of (a) speciation, (b) character change, and (c) extinction under different degrees of asymmetry in character change rates with corresponding tip ratios.** Point of intersection between red lines represents known values.

Estimates of asymmetries in character change are not as accurate or precise as estimating rates of speciation (Figure 4b). In general, precision decreases as the rate difference increase results in a high tip bias scenario, with parameter estimation being poor for a known estimate of a 40× rate difference in character change. Symmetrical speciation rates (λ_0_**=** 0.1, λ_1_**=** 0.1) are well estimated when the rate of character change is 2× (low tip bias) as seen in Figure 4a. However, with a 40× (high tip bias) difference in the rate of character change the precision of parameter estimation appears to decrease, and the number of estimates for highly asymmetrical speciation rates increases (Figure 4a). Parameter estimation of known extinction rates (*μ*_0_**=** 0.03, *μ*_1_**=** 0.03) is more accurate under the 2× (low tip bias) scenario rather than the 40× (high tip bias) scenario where estimates of known extinction values are very poor (Figure 4c).

#### Estimating parameters under asymmetrical extinction

**Figure 5:**
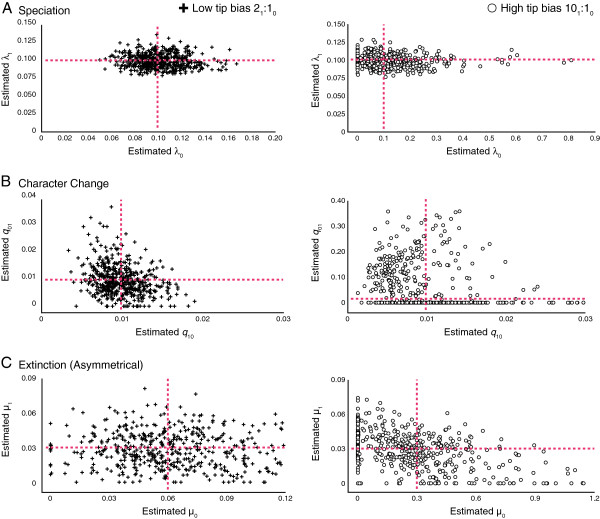
**Parameter estimations of (a) speciation, (b) character change, and (c) extinction under different degrees of asymmetry in extinction rates with corresponding tip ratios.** Point of intersection between red lines represents known values.

Estimates of known extinction values are poor and seem to lack precision, with precision decreasing as the difference in extinction rates increase (Figure 5c). Speciation values are well estimated to the known values (λ_0_**=** 0.1, λ_1_**=** 0.1) when the difference between extinction rates is 2× (low tip bias), but accuracy and precision seems to decrease dramatically as the extinction rate asymmetry increases (high tip bias), leading to an abundance of highly asymmetrical estimates of speciation rates (Figure 5a). Parameter estimation of character change has the same pattern, in which the known rate (*q*_01_**=** 0.01, *q*_10_**=** 0.01) is well estimated under 2× (low tip bias) extinction rate differences (Figure 5b), but is poorly estimated under an increased difference in extinction rate asymmetry that leads to high tip bias (Figure 5b). This poor estimation leads to a dramatic increase in asymmetrical rates of character change that favor transitions from State 0 to 1.

## Discussion

### Impact of tree size on power

The statistical power of the BiSSE method depends on the number of taxa and the degree of asymmetry in rates of speciation, extinction, and character state change. In terms of tree size, BiSSE achieves extremely low power when testing hypotheses of rate asymmetry if fewer than 100 taxa are used in the analysis, even when rates are known to be highly asymmetrical (Figure 1). As a result, the potential for a Type II error (failing to reject the null hypothesis when the alternate hypothesis is true) is extremely high.

The highest power attributed to any rate asymmetry associated with 300 taxa is only 50% (Figure 1) under a six parameter model. Researchers that attempt to utilize the BiSSE method with fewer than 300 taxa should take caution. Below 200 taxa there is little statistical power associated with identifying rate asymmetries, regardless of whether strong asymmetries exist. Maddison et al. [1] hypothesized that large amounts of data would be needed to distinguish significant asymmetries because there are many ways to arrive at a given phylogeny.

### Power of simplified models

If external information justifies a simplification of the model, then greater power can be achieved. We found an overall increase in power when utilizing a four parameter model over a six parameter model regardless of which rate possesses the asymmetry (Figure 2, Additional file 1: Table S4), although tree sizes greater than 300 tips are still desirable. Whether researchers can take advantage of this greater power using a model with fewer free parameters depends of course on whether it is reasonable to assume in advance that any asymmetries are restricted to one rate.

### Confounding processes

Strong asymmetries in rates of speciation, character state change, and extinction yielded, as expected, a strong excess of tips with a single character state (Table 1). The question to a biologist observing such a pattern is; which rate’s asymmetry might have led to such excess? Maddison [20] hypothesized that teasing apart parameters that are the cause of taxonomic state frequency asymmetry is difficult, and that simultaneously estimating these parameters may help address this issue. We found that under best-case scenarios where high tip bias is absent and sample size is high (preferably greater than 300 taxa), parameters are estimated accurately and precisely (Figures 3, 4, 5, Additional file 2: Figure S1, Table 2). This indicates that the BiSSE method is capable of identifying the rate asymmetry that is causing taxonomic excess when datasets are used with a sufficient amount of tips and where high tip bias is absent, as seen in Figures 3, 4, 5 (Table 2). In these scenarios, BiSSE is able to properly identify the known process with the rate asymmetry (Table 2), whether it was speciation (Figure 3a), character change (Figure 4b), or extinction (Figure 5c). This is further corroborated by the power, which remains high in cases where sample size is large and tip ratio bias is minimized (Figure 1). However, teasing apart which rate asymmetries are causing taxonomic excess of one character state is a problem under two conditions; if tree size is small (Additional file 2: Figure S1), regardless of the degree of tip bias, and if there is a high degree of tip ratio bias towards one state, even while simultaneously estimating parameters using the BiSSE method with trees of substantial size (500 tips, Figures 3, 4, 5, Additional file 2: Figure S1, Table 2). Below we discuss why high tip ratio bias leads to a decrease in power and parameter estimation, and how much tip ratio bias is necessary to have an adverse affect on teasing apart confounding processes.

**Table 1:** Parameter values for rate asymmetry simulations used to assess power and parameter estimation of BiSSE method

**Rate asymmetry**	**Tip ratio**	**Figure**	**% State 0**
Speciation (*q*_01_ and *q*_10_ = 0.01, *μ*_0_ and *μ*_1_ = 0.03)			
1.25× (λ_0_**=** 0.1, λ_1_**=** 0.125)	3_1_:1_0_	1A	29.23
1.5× (λ_0_**=** 0.1, λ_1_**=** 0.15)	5_1_:1_0_	1A	19.33
2× (λ_0_**=** 0.1, λ_1_**=** 0.2)	10_1_:1_0_	1A	9.90
3× (λ_0_**=** 0.1, λ_1_**=** 0.3)	20_1_:1_0_	1A	4.94
4× (λ_0_**=** 0.1, λ_1_**=** 0.4)	30_1_:1_0_	1A	3.19
5× (λ_0_**=** 0.1, λ_1_**=** 0.5)	40_1_:1_0_	1A	2.50
10× (λ_0_**=** 0.1, λ_1_**=** 1.0)	90_1_:1_0_	1A	1.13
20× (λ_0_**=** 0.1, λ_1_**=** 2.0)	180_1_:1_0_	1A	0.51
Character Change (λ_0_ and λ_1_= 0.1, *μ*_0_ and *μ*_1_ = 0.03)			
2× (*q*_01_**=** 0.01, *q*_10_**=** 0.005)	2_1_:1_0_	1B	33.96
3× (*q*_01_**=** 0.015, *q*_10_**=** 0.005)	3_1_:1_0_	1B	24.01
4× (*q*_01_**=** 0.02, *q*_10_**=** 0.005)	4_1_:1_0_	1B	20.57
5× (*q*_01_**=** 0.025, *q*_10_**=** 0.005)	5_1_:1_0_	1B	16.69
10× (*q*_01_**=** 0.05, *q*_10_**=** 0.005)	10_1_:1_0_	1B	9.14
20× (*q*_01_**=** 0.1, *q*_10_**=** 0.005)	20_1_:1_0_	1B	4.69
40× (*q*_01_**=** 0.2, *q*_10_**=** 0.005)	40_1_:1_0_	1B	2.39
Extinction (λ_0_ and λ_1_= 0.1, *q*_01_ and *q*_10_ = 0.01)			
2× (*μ*_0_**=** 0.06, *μ*_1_**=** 0.03)	3_1_:1_0_	1C	23.85
3× (*μ*_0_**=** 0.09, *μ*_1_**=** 0.03)	6_1_:1_0_	1C	13.21
4× (*μ*_0_**=** 0.12, *μ*_1_**=** 0.03)	9_1_:1_0_	1C	9.29
5× (*μ*_0_**=** 0.15, *μ*_1_**=** 0.03)	12_1_:1_0_	1C	7.12
10× (*μ*_0_**=** 0.3, *μ*_1_**=** 0.03)	27_1_:1_0_	1C	3.40

The observed percent of terminals with State 0 are for simulations with 500 taxa. Observed tip ratios were nearly identical for other taxa sizes (50, 100, and 300).

**Table 2:** Summary statistics of parameter estimates for rate asymmetry simulations with 500 taxa under low and high tip bias scenarios

**Rate asymmetry**	**λ**_**0**_	**λ**_**1**_	***q***_**10**_	***q***_**01**_	***μ***_**0**_	***μ***_**1**_
**Speciation (Figure**3**)**						
Low Tip Bias (3_1_:1_0_)						
Simulated	0.1	0.125	0.01	0.01	0.03	0.03
Estimated (Mean ± S_*e*_)	0.101 ± 0.018	0.125 ± 0.013	0.01 ± 0.003	0.01 ± 0.006	0.035 ± 0.031	0.029 ± 0.022
Medium Tip Bias (40_1_:1_0_)						
Simulated	0.1	0.5	0.01	0.01	0.03	0.03
Estimated (Mean ± S_*e*_)	0.107 ± 0.119	0.502 ± 0.033	0.012 ± 0.007	0.09 ± 0.295	0.103 ± 0.33	0.034 ± 0.046
High Tip Bias (180_1_:1_0_)						
Simulated	0.1	2.0	0.01	0.01	0.03	0.03
Estimated (Mean ± S_*e*_)	0.377 ± 2.57	2.048 ± 0.124	0.018 ± 0.028	3.1 ± 7.6	1.78 ± 4.85	0.11 ± 0.17
**Character Change (Figure**4**)**						
Low Tip Bias (2_1_:1_0_)						
Simulated	0.1	0.1	0.005	0.01	0.03	0.03
Estimated (Mean ± S_*e*_)	0.103 ± 0.033	0.099 ± 0.011	0.005 ± 0.002	0.0099 ± 0.005	0.038 ± 0.053	0.03 ± 0.02
Medium Tip Bias (10_1_:1_0_)						
Simulated	0.1	0.1	0.005	0.05	0.03	0.03
Estimated (Mean ± S_*e*_)	0.104 ± 0.032	0.098 ± 0.009	0.0058 ± 0.006	0.057 ± 0.218	0.048 ± 0.075	0.026 ± 0.014
High Tip Bias (40_1_:1_0_)						
Simulated	0.1	0.1	0.005	0.2	0.03	0.03
Estimated (Mean ± S_*e*_)	0.142 ± 0.145	0.098 ± 0.008	0.008 ± 0.009	0.193 ± 0.443	0.281 ± 0.462	0.023 ± 0.014
**Extinction (Figure**5**)**						
Low Tip Bias (2_1_:1_0_)						
Simulated	0.1	0.1	0.01	0.01	0.06	0.03
Estimated (Mean ± S_*e*_)	0.1 ± 0.02	0.099 ± 0.01	0.009 ± 0.002	0.009 ± 0.006	0.063 ± 0.031	0.028 ± 0.016
Medium Tip Bias (3_1_:1_0_)						
Simulated	0.1	0.1	0.01	0.01	0.09	0.03
Estimated (Mean ± S_*e*_)	0.077 ± 0.014	0.102 ± 0.009	0.009 ± 0.003	0.012 ± 0.013	0.093 ± 0.047	0.027 ± 0.014
High Tip Bias (10_1_:1_0_)						
Simulated	0.1	0.1	0.01	0.01	0.3	0.03
Estimated (Mean ± S_*e*_)	0.116 ± 0.109	0.098 ± 0.008	0.013 ± 0.03	0.194 ± 1.2	0.29 ± 0.33	0.028 ± 0.015

When taxa are saturated for a particular state (high tip ratio bias), the BiSSE method estimates high rate asymmetries to explain this pattern, even for those rates known to be low and symmetrical (Figure 3, 4, 5, Table 2). For example, when extinction is 10× higher for State 0 than State 1 (Tip bias of 10_1_:1_0_), the extinction asymmetry is detected, but the rate of character change is also estimated to be highly asymmetrical (Table 2). The method infers rapid change from State 0 to 1 (Figure 5b, Table 2) when in fact, the constrained rates were fairly low and symmetrical (*q*_01_**=** 0.01, *q*_10_**=** 0.01). The inability to identify confounding processes when there is high tip ratio bias results in the observed decrease in power when the degree of rate asymmetry increases in either the rate of speciation, extinction, or character change (Figure 1). This issue is not resolved with larger tree sizes (Figure 1), and the degree of tip bias needed to adversely affect the accuracy and precision of parameter estimation varied with the process (e.g., speciation, extinction, character change).

The amount of bias needed to reduce accuracy and precision of parameter estimation, leading to worst-case scenarios, changed depending on the process. When estimating parameters associated with asymmetries of speciation rates, power sharply decreases when fewer than 2.5% of the taxa have one of the binary traits (Tables 1, 2, Figures 1, 3). Character change and extinction rate asymmetries are more adversely affected by trait rarity, with the worst-case scenarios happening when a binary trait occurs in fewer than 8–10% of the terminal taxa. As with speciation, high tip ratio bias leads to a sharp decrease in power regardless of sample size (Figures 1b, 1c, 4, 5; Tables 1, 2, Additional file 1: Tables S1-S3). Overall, caution is recommended when using the BiSSE method if tip ratio bias is greater than a 10:1 ratio for binary states in terminal taxa, as this level of trait rarity in one state may have a negative impact on the power of the analysis (regardless of sample size) and the ability to identify confounding processes under these worst-case scenarios.

The observed pattern of a decrease in power associated with speciation, extinction, and character change when rates become increasingly asymmetrical is troubling (Figure 1). In general, investigators should be cautious using the BiSSE method when one of the binary characters in question is exceedingly rare in their data sets (less than 10%). BiSSE may mistakenly estimate the wrong parameter (or combination of parameters) to be the cause of taxonomic excess in situations where high tip ratio bias is observed, and increased tree size does not alleviate this issue. If investigators report a significant result, there is a possibility that the inability to identify confounding processes in these scenarios may impact the interpretation of the causes for the observed pattern of state asymmetries. Further work is needed to establish a robust methodology within the BiSSE framework for teasing apart the parameters that are directly contributing to character state bias under these high tip ratio scenarios.

### Difference in estimation accuracy among processes of change

We found that the BiSSE method is far more accurate and precise with estimates of rates of speciation and character change than with extinction rates (Table 2, Figures 3, 4, 5, Additional file 2: Figure S1), which was also noted by Maddison et al. [1]. Overall, a greater degree of tip ratio bias is needed to reduce the accuracy and precision of speciation parameter estimation and a loss of power when the rate asymmetry is related to speciation (discussed previously). While extinction can leave a signal in molecular phylogenetic trees recovered from extant taxa alone [21,22], estimating extinction rates from molecular phylogenies often results in rates approaching zero, which is potentially the result of cladogenetic events being directly inferred from molecular phylogenies and not extinction events [22]. Recently, Rabosky [23] indicated that without information from the fossil record, estimating extinction rates from molecular data alone may be potentially impossible if rates of diversification vary across a topology. As demonstrated by this study, BiSSE is capable of estimating rates of extinction from known values given sufficient data (e.g., large tree size, low tip bias), albeit with far less accuracy and precision than rates of speciation or character change. Also, if an asymmetry in extinction rates leads to a high tip ratio bias, the accuracy and precision of extinction rates estimation decreases (Figure 5c). Finally, the accuracy and precision of estimating rates is also impacted by a small sample size (>300 tips) regardless of tip ratio bias as indicated in Additional file 2: Figure S1. In addition to low power associated with small tree sizes, investigators should be cautious of a significant result if tree size is less than 300 tips, as the inferred cause(s) for the potential evolutionary pattern may be misled by the issue of confounding processes.

### Identifying critical values

An additional issue uncovered in this analysis is the difficulty in finding appropriate critical values. The critical values from our simulations suffer from a great deal of variation (Additional file 1: Tables S1-S4). The nearly consistent difference in critical values suggests that simply comparing the statistic to a χ^2^ distribution may not be appropriate as suggested by Maddison et al. [1]. Therefore, we suggest simulating your own critical values, using as many replicates as possible, as we did here. Suitable simulators are available in both Mesquite and the Diversitree R-package [2].

### Implementation issues

Another possibility is that the method performs better than these results suggest, but limitations of our likelihood optimizer limit the power of our implementation. Our implementation, as described in Maddison et al. [1] uses several techniques to overcome the limitations of the Brent [24] optimizer, including using multiple searches from randomly generated starting points, and the option of starting searches from values estimated from more constrained models. Tests (Midford unpublished) with alternative optimizers, using the Diversitree package described FitzJohn et al. [2] indicate that little, if any, loss of power is actually related to the choice of optimizer.

## Conclusion

The power of the BiSSE likelihood method to test hypotheses of rate asymmetry is susceptible to both tree size and variation in parameter rates. Overall, power of the BiSSE method is low if the tree size is below 300 taxa, and investigators should take special care to investigate the power of their results if applying the BiSSE method to topologies with fewer than 300 tips. Power is increased when estimating fewer parameters, so utilizing a four parameter model to test hypotheses may be preferable if appropriate.

This study indicates that contrary to the hope expressed in Maddison [20], the problem of confounding effects can still occur while estimating process parameters simultaneously if there is low sample size and/or high tip ratio bias. Under scenarios of large sample size (greater than 300 taxa) and low tip bias, the BiSSE method accurately and precisely identifies the rate parameters contributing to the observed taxonomic excess. However, if diversification rate parameters are too asymmetrical (yielding a high tip ratio bias) and/or sample size is low, BiSSE is unable to accurately estimate rates. This in turn results in a dramatic decrease of power. We recommend that investigators must be cautious when interpreting their results if there is a character state bias among tips greater than a 10:1 ratio in favor of either binary state. In these worst-case situations, properly identifying the process responsible for taxonomic excess may be impossible regardless of the number of tips in the dataset. If investigators using data with fewer than 300 tips and/or with high tip bias report a significant result, there exists a possibility that the issue of confounding effects has misled the identified rate cause(s) of the significant result. Further work is needed within the BiSSE framework to develop methods to better identify which parameters are causing the character state bias in these worst-case scenarios (e.g., low sample size, high tip bias). Further exploration of the impact of multiple rate asymmetries is also needed. However, it is clear that if multiple rate asymmetries are occurring that promote high tip ratio bias there will be difficulties with power and parameter estimation.

## Methods

### Hypothesis testing and the power of the BiSSE method

The BiSSE likelihood calculation and parameter estimations were done in the Diverse package [25] of Mesquite 2.7 [26]. Maddison et al. [1] suggested that the probability of rejecting a false null hypothesis (power) may vary with the number of species in an analysis, and with the degree of rate difference among parameters. The initial exploration of power for the BiSSE method in Maddison et al. [1] focused on three rate asymmetric scenarios (one for speciation, character change, and extinction) with a tree size of 500 tips. To explore the full range of power for the BiSSE method when using the six parameter model, 500 trees were simulated under a variety of tip sizes and parameter combinations where an asymmetry in one rate parameter was introduced (e.g., λ_0_ > λ_1_).

When a tree and character are simulated with asymmetrical rates in one process — speciation, extinction, or character state change — our question was whether the BiSSE method could detect this asymmetry and estimate the rates correctly. A biologist facing such a question with real data may be interested in just one of the processes (e.g., is there an asymmetry in speciation rates?), and would therefore face a choice: are the other processes assumed to be symmetrical (i.e. extinction rates μ_0_ = μ_1_) or not? Assuming the other processes to be symmetrical reduces the complexity of the models and permits the method to focus entirely on the process of interest. We studied the benefits of such simplifying assumptions as described in the next section. However, because biologists typically would not have information confirming the other processes to be symmetrical, in most of our analyses we permitted all three processes to be asymmetrical, thus requiring us to compare the null five-parameter model against a full six parameter model.

Thus, for any given asymmetry simulation, the BiSSE likelihood score for the full six parameter model was calculated and compared to the likelihood score of a corresponding five parameter model where the rate parameter with an asymmetry being tested is constrained to be equal for both states (e.g., λ_0_ = λ_1_). In addition, a null hypothesis set of simulations with 500 trees was generated where all rate values are symmetrical, and a distribution of the BiSSE likelihood score difference for the six and five parameter models were calculated for each null hypothesis. Power was determined as the percentage of likelihood difference scores between the six and five parameter models of the asymmetrical simulations that were above the 5% cutoff value established by the corresponding likelihood difference score distributions of the rate symmetrical null hypothesis simulations. We simulated sets of 500 trees with varying bias for each of the three parameter pairs as well as sets without bias and each rate parameter combination (Table 1) was tested under tree sizes of 50, 100, 300, and 500 taxa, respectively, in which the probability of the root state was stationary [27].

Many asymmetries in rates would yield biases in the frequency of the two character states observed in the contemporaneous species at the tips of the tree, with stronger asymmetries yielding strong biases. Insofar as BiSSE’s behavior might differ depending on the strength of the bias in state frequencies, we explored examples with rate parameter asymmetries that would result in a range of observed high and low biases in character state distributions across the tips (Tables 1 and 2). Ratios of tip bias (taxa with state 0 relative to state 1) were calculated from their corresponding biases in the model rates using the stationary frequency formula in Appendix 2 of Maddison et al. [1].

(1) $$g\;xˆ\left(1-xˆ\right)-xˆq01+\left(1-xˆ\right)q10=0$$

Where *g* = λ_0_ – μ_0_ – λ_1_+μ_1_, and *x*ˆ is the stationary frequency of state 0.

As expected, tip bias increased as asymmetries in the simulation parameters increased steadily across all tree sizes, and the observed asymmetry in taxa with each state matched expectations given the starting rate asymmetries (Table 1, Additional file 1: Tables S1, S2, and S3).

#### Power in reduced (4-parameter) models

We investigated whether estimating fewer parameters would lead to an increase in power. In some biological systems it may be reasonable to assume from the beginning that some processes are symmetrical. To examine this we compared the results of the previously described 5 *vs.* 6 parameter tests with the results of tests involving reduced models of 3 *vs.* 4 parameters. The reduced test compared a four-parameter model, where two of the rates were constrained to be equal in both character states, for example (λ_0_, λ_1_, μ_0_=μ_1_, *q*_01_=*q*_10_), and a three-parameter model where all three rates were constrained to be equal in both character states. These scenarios were done for two tip bias scenarios, one with a small bias (3:1 character state ratio) and one with a greater degree of bias (7:1 character state ratio) as seen in Additional file 1: Table S4. We simulated sets of 500 trees with both levels of bias for each of the three parameter pairs as well as sets without bias for a six/five and four/three model comparisons as seen in Additional file 1: Table S4.

### Estimating parameters in asymmetrical scenarios

Using BiSSE to estimate rates of speciation, extinction and character change may be illuminating not only to understand the degree of any asymmetries, but also to distinguish which potential factor (biased speciation, extinction, or character change) is responsible for an observed excess of species with a particular state. Rate parameters for unconstrained (six parameter) models were tabulated under a best-case scenario representing a small degree of tip bias and a worst-case scenario that included a high degree of tip bias with tree sizes of 500 taxa. Parameters were estimated from the same 500 trees and respective characters that were used to calculate the BiSSE likelihood difference (Table 1). With these simulated cases we asked whether the parameter values were estimated well, with a special focus on whether a bias in one process (e.g., extinction) might be confounded with a bias in another process (e.g., character change).

### Implementation

The computer software used in this study was a refined version of the package Diverse [25] described in Maddison et al. [1], with these refinements already implemented in Mesquite [26]. Two of these refinements are described here. The simulation module (“Evolving Binary Speciation/Extinction Character”) was modified to generate trees more efficiently by means of a continuous approximation. The updated module calculates the rate of events on the tree as the product of the number of terminal branches on the tree and sum of rates for each event type. The time to the next event was drawn from an exponential distribution and the type and location (branch) of the new event were drawn from appropriately weighted uniform distributions. Following this, all terminal branches were extended to the time point of the generated event. This process continued until the tree reached a limiting number of tips, or the unlikely event that all terminal branches became extinct. The second refinement is an enhanced parameter estimator that uses a numerical integrator that implements the RKF45 method [28]. The RKF45 method improved on the RK4 method, used in Maddison et al. [1] by adaptively adjusting the step-size used in the integration process. Our implementation specified a starting step-size and subsequent changes in step-size were limited a range of 1/10x to 10x the original step-size.

## Competing interests

The authors declare that they have no competing interests.

## Authors’ contributions

MPD and PEM designed the study, carried out simulations, and performed the analyses. All authors interpreted the results, wrote the manuscript, and approved the final version of the manuscript.

## Supplementary Material

Additional file 1: Table S1 Power of asymmetrical speciation rate simulations. Remaining parameters were symmetrical for each simulation (*q*_01_**=** 0.01, *q*_10_**=** 0.01, *μ*_0_**=** 0.03, *μ*_1_**=** 0.03). Power is plotted in Figure 1A. The observed percent of terminal taxa with State 0 is indicated by the mean value from 500 simulations. **Table S2**. Power of simulations for character rate change. Remaining parameters were symmetrical for each simulation (*μ*_0_**=** 0.03, *μ*_1_**=** 0.03, λ _0_**=** 0.1, λ _1_**=** 0.1). Power is plotted in Figure 1B. The observed percent of terminal taxa with State 0 is indicated by the mean value from 500 simulations. **Table S3**. Power of asymmetrical extinction rate simulations. Remaining parameters were symmetrical for each simulation (*q*_01_**=** 0.01, *q*_10_**=** 0.01, λ _0_**=** 0.1, λ _1_**=** 0.1). Power is plotted in Figure 1C. The observed percent of terminal taxa with State 0 is indicated by the mean value from 500 simulations. **Table S4**. This table lists statistical power of the BiSSE model for 500 simulations containing 3:1 and 7:1 biases in terminal states for varying tree sizes for likelihood comparisons of power in four versus six parameter models. Power is plotted in Figure 2. Using the stationary frequency formula, in an iterative calculation, we obtained ratios of rates necessary to generate a low bias representative (3_1_:1_0_) and high bias representative (7_1_:1_0_) tip ratios using values symmetrically placed around base rates (λ = 0.1, μ = 0.05, and q = 0.005). For the low bias, rate ratios were 1.1425, 1.3046 and 3.0 for speciation, extinction and character change respectively, yielding simulated rates for speciation λ_0_=0.0936, λ_1_**=** 0.10689, for extinction, μ_0_=0.04378, μ_1_**=** 0.05711, and for character change *q*_0_= 0.00289, *q*_1_**=** 0.00866. For the high bias, rate ratios were 1.407, 1.960, and 7.000, yielding simulated rates for speciation λ_0_=0.0843, λ_1_**=**0.1186, for extinction μ_0_=0.0357, μ_1_**=** 0.07, and for character change *q*_0_=0.00189, *q*_1_**=** 0.01323. Simulated rates without bias were set to their base rates. (DOCX 36 kb) Click here for file

Additional file 2: Figure S1 Parameter estimations of (**a**) speciation, (**b**) character change, and (**c**) extinction under different tree sizes and degrees of asymmetry in speciation rates with corresponding tip ratios. Point of intersection between red lines represents known values. (PDF 640 kb) Click here for file

## References

[B1] Maddison WP Midford PE Otto SP Estimating a binary character’s effect on speciation and extinction Syst Biol 2007 56 701 710 10.1080/10635150701607033 17849325

[B2] FitzJohn RG Maddison WP Otto SP Estimating trait-dependent speciation and extinction rates from incompletely resolved phylogenies Syst Biol 2009 58 595 611 10.1093/sysbio/syp067 20525612

[B3] Owens IPF Bennet PM Harvey PH Species richness among birds: body size, life history, sexual selection or ecology Proc R Soc Lond B 1999 266 933 939 10.1098/rspb.1999.0726

[B4] Magallón S Sanderson MJ Absolute diversification rates in angiosperm clades Evol 2001 55 1762 1780 10.1111/j.0014-3820.2001.tb00826.x 11681732

[B5] Vamosi SM Vamosi JC Endless tests: guidelines for analyzing non-nested sister group comparisons Evol Ecol Res 2005 7 567 579

[B6] FitzJohn RG Quantitative traits and diversification Syst Biol 2010 59 619 633 10.1093/sysbio/syq053 20884813

[B7] Goldberg EE Lancaster LT Ree RH Phylogenetic inference of reciprocal effects between geographic range evolution and diversification Syst Biol 2011 60 451 465 10.1093/sysbio/syr046 21551125

[B8] Goldberg EE Igic B On phylogenetic tests of irreversible evolution Evol 2008 62 2727 2741 10.1111/j.1558-5646.2008.00505.x 18764918

[B9] Alfaro ME Brock CD Barbury BL Wainwright PC Does evolutionary innovation in pharyngeal jaws lead to rapid lineage diversification in labrid fishes? BMC Evol Biol 2009 9 255 10.1186/1471-2148-9-255 19849854 PMC2779191

[B10] Armbruster SW Pelabon C Hansen TF Bolstad GH Macroevolutionary patterns of pollination accuracy: a comparison of three genera New Phy 2009 183 600 617 10.1111/j.1469-8137.2009.02930.x 19594697

[B11] Lynch VJ Live-birth in vipers (Viperidae) is a key innovation and adaptation to global cooling during the Cenozoic Evol 2009 63 2457 2465 10.1111/j.1558-5646.2009.00733.x 19563326

[B12] Anacker BL Whittall JB Goldberg EE Harrison SP Origins and consequences of serpentine endemism in the California flora Evol 2010 65 365 376 10.1111/j.1558-5646.2010.01114.x 20812977

[B13] Lynch VJ Wagner GP Did egg-laying boas break dollo’s law? Phylogenetic evidence for reversal to oviparity in sand boas (Eryx: Boidae) Evol 2010 64 207 216 10.1111/j.1558-5646.2009.00790.x 19659599

[B14] Price SA Wainwright PC Bellwood DR Kazancloglu E Collar DC Near TJ Functional innovations and morphological diversification in parrotfish Evol 2010 64 3057 3068 10.1111/j.1558-5646.2010.01036.x 20497217

[B15] Rabosky DL Glor RE Equilibrium speciation dynamics in a model adaptive radiation of island lizards PNAS 2010 107 22178 22183 10.1073/pnas.1007606107 21135239 PMC3009809

[B16] Stireman JO Devlin H Carr TG Abbot P Evolutionary diversification of the gall midge genus Asteromyia (Cecidomyiidae) in a multitrophic ecological context Mol Phyl Evol 2010 54 194 210 10.1016/j.ympev.2009.09.010 19765662

[B17] Johnson TJ FitzJohn RG Smith SD Rausher MD Otto SP Loss of sexual recombination and segregation is associated with increased diversification in evening primroses Evol 2011 65 3230 3240 10.1111/j.1558-5646.2011.01378.x PMC3202692 22023588

[B18] Wiens JJ Re-evolution of lost mandibular teeth in frogs after more than 200 million years, and re-evaluating dollo’s law Evol 2011 65 1283 1296 10.1111/j.1558-5646.2011.01221.x 21521189

[B19] Wilson AW Binder M Hibbett DS Effects of gasteroid fruiting body morphology on diversification rates in three independent clades of fungi estimated using binary state speciation and extinction analysis Evol 2011 65 1305 1322 10.1111/j.1558-5646.2010.01214.x 21166793

[B20] Maddison WP Confounding asymmetries in evolutionary diversification and character change Evol 2006 60 1743 1746 17017073

[B21] Nee S Holmes EC May RM Harvey PH Extinction rates can be estimated from molecular phylogenies Phil Trans R Soc Lond B 1994 344 305 311 10.1098/rstb.1994.0068 8878259

[B22] Paradis E Statistical analysis of diversification with species traits Evol 2005 59 1 12 15792222

[B23] Rabosky DL Extinction rates should not be estimated from molecular phylogenies Evol 2010 64 1816 1824 10.1111/j.1558-5646.2009.00926.x 20030708

[B24] Brent RP Algorithms for optimization without derivatives 1973 New Jersey: Prentice Hall

[B25] Midford P Maddison WP Diverse package for mesquite. Version 1.0. 2007 http://mesquiteproject.org

[B26] Maddison WP Maddison DR Mesquite: a modular system for evolutionary analysis. Version 2.7 2009 http://mesquiteproject.org

[B27] Felsenstein J Evolutionary trees from DNA sequences: a maximum likelihood approach J Mol Evol 1981 17 368 376 10.1007/BF01734359 7288891

[B28] Johnson LW Riess DR Numerical analysis 1982 Massachusetts: Addison-Wesley Publishing Company

