# Supplementary material for: Exploring power and parameter estimation of the BiSSE method for analyzing species diversification
Source: BMC Evol Biol. 2013 Feb 11;13:38. doi: 10.1186/1471-2148-13-38 (PMC3583807; doi:10.1186/1471-2148-13-38)
Supplement: Additional file 1: Table S1 — Power of asymmetrical speciation rate simulations. Remaining parameters were symmetrical for each simulation (q01= 0.01, q10= 0.01, μ0= 0.03, μ1= 0.03). Power is plotted in Figure 1A. The observed percent of terminal taxa with State 0 is indicated by the mean value from 500 simulations. Table S2. Power of simulations for character rate change. Remaining parameters were symmetrical for each simulation (μ0= 0.03, μ1= 0.03, λ 0= 0.1, λ 1= 0.1). Power is plotted in Figure 1B. The observed percent of terminal taxa with State 0 is indicated by the mean value from 500 simulations. Table S3. Power of asymmetrical extinction rate simulations. Remaining parameters were symmetrical for each simulation (q01= 0.01, q10= 0.01, λ 0= 0.1, λ 1= 0.1). Power is plotted in Figure 1C. The observed percent of terminal taxa with State 0 is indicated by the mean value from 500 simulations. Table S4. This table lists statistical power of the BiSSE model for 500 simulations containing 3:1 and 7:1 biases in terminal states for varying tree sizes for likelihood comparisons of power in four versus six parameter models. Power is plotted in Figure 2. Using the stationary frequency formula, in an iterative calculation, we obtained ratios of rates necessary to generate a low bias representative (31:10) and high bias representative (71:10) tip ratios using values symmetrically placed around base rates (λ = 0.1, μ = 0.05, and q = 0.005). For the low bias, rate ratios were 1.1425, 1.3046 and 3.0 for speciation, extinction and character change respectively, yielding simulated rates for speciation λ0=0.0936, λ1= 0.10689, for extinction, μ0=0.04378, μ1= 0.05711, and for character change q0= 0.00289, q1= 0.00866. For the high bias, rate ratios were 1.407, 1.960, and 7.000, yielding simulated rates for speciation λ0=0.0843, λ1=0.1186, for extinction μ0=0.0357, μ1= 0.07, and for character change q0=0.00189, q1= 0.01323. Simulated rates without bias were set to their base rates. (DOCX 36 kb) [file 1471-2148-13-38-S1.docx]

| Rate of Speciation | 5% Cutoff | Power | Observed State 0 |
| --- | --- | --- | --- |
| 1.25× (λ_0_ **=** 0.1, λ_1_**=** 0.125; Ratio 3_1_:1_0_; Expected State 0 = 25%)  500 taxa  300 taxa  100 taxa  50 taxa  1.5× (λ_0_ **=** 0.1, λ_1_**=** 0.15; Ratio 5_1_:1_0_; Expected State 0 = 16.7%)  500 taxa  300 taxa  100 taxa  50 taxa  2× (λ_0_ **=** 0.1, λ_1_**=** 0.2; Ratio 10_1_:1_0_; Expected State 0 = 9%)  500 taxa  300 taxa  100 taxa  50 taxa  3× (λ_0_ **=** 0.1, λ_1_**=** 0.3; Ratio 20_1_:1_0_; Expected State 0 = 4.8%)  500 taxa  300 taxa  100 taxa  50 taxa  4× (λ_0_ **=** 0.1, λ_1_**=** 0.4; Ratio 30_1_:1_0_; Expected State 0 = 3.2%)  500 taxa  300 taxa  100 taxa  50 taxa  5× (λ_0_ **=** 0.1, λ_1_**=** 0.5; Ratio 40_1_:1_0_; Expected State 0 = 2.4%)  500 taxa  300 taxa  100 taxa  50 taxa  10× (λ_0_ **=** 0.1, λ_1_**=** 1.0; Ratio 90_1_:1_0_; Expected State 0 = 1.1%)  500 taxa  300 taxa  100 taxa  50 taxa  20× (λ_0_ **=** 0.1, λ_1_**=** 2.0; Ratio 180_1_:1_0_; Expected State 0 = 0.55%)  500 taxa  300 taxa  100 taxa  50 taxa | 1.875  2.107  2.818  4.575  1.875  2.107  2.818  4.575  1.875  2.107  2.818  4.575  1.875  2.107  2.818  4.575  1.875  2.107  2.818  4.575  1.875  2.107  2.818  4.575  1.875  2.107  2.818  4.575  1.875  2.107  2.818  4.575 | 20.88%  11.20%  8.60%  3.21%  40.72%  25.85%  14.60%  3.20%  57.00%  42.40%  12.60%  2.60%  72.40%  47.20%  12.00%  1.80%  69.00%  49.00%  9.80%  1.80%  71.00%  42.80%  10.20%  1.80%  53.60%  24.40%  2.60%  1.40%  29.80%  8.15%  1.20%  1.00% | 29.23%  28.74%  30.86%  28.39%  19.33%  18.85%  10.47%  19.26%  9.90%  10.79%  11.60%  11.24%  4.94%  4.64%  5.02%  4.85%  3.19%  3.38%  3.36%  4.72%  2.50%  2.55%  2.84%  3.04%  1.13%  3.05%  1.28%  1.07%  0.51%  0.54%  0.51%  0.86% |

**Table S1:** Power of asymmetrical speciation rate simulations. Remaining parameters were symmetrical for each simulation (*q*_01_ **=** 0.01, *q*_10_ **=** 0.01, *μ*_0_ **=** 0.03, *μ*_1_ **=** 0.03). Power is plotted in Fig 1A. The observed percent of terminal taxa with State 0 is indicated by the mean value from 500 simulations.

| 2× (q_01_ **=** 0.01, q_10_ **=** 0.005; Ratio 2_1_:1_0_; Expected State 0 = 33.33%)  500 taxa  300 taxa  100 taxa  50 taxa  3× (q_01_ **=** 0.015, q_10_ **=** 0.005; Ratio 3_1_:1_0_; Expected State 0 = 25%)  500 taxa  300 taxa  100 taxa  50 taxa  4× (q_01_ **=** 0.02, q_10_ **=** 0.005; Ratio 4_1_:1_0_; Expected State 0 = 20%)  500 taxa  300 taxa  100 taxa  50 taxa  5× (q_01_ **=** 0.025, q_10_ **=** 0.005; Ratio 5_1_:1_0_; Expected State 0 = 16.66%)  500 taxa  300 taxa  100 taxa  50 taxa  10× (q_01_ **=** 0.05, q_10_ **=** 0.005; Ratio 10_1_:1_0_; Expected State 0 = 9.1%)  500 taxa  300 taxa  100 taxa  50 taxa  20× (q_01_ **=** 0.1, q_10_ **=** 0.005; Ratio 20_1_:1_0_; Expected State 0 = 4.7%)  500 taxa  300 taxa  100 taxa  50 taxa  40× (q_01_ **=** 0.2, q_10_ **=** 0.005; Ratio 40_1_:1_0_; Expected State 0 = 2.43%)  500 taxa  300 taxa  100 taxa  50 taxa | 2.149  2.048  2.379  5.661  2.149  2.048  2.379  5.661  2.149  2.048  2.379  5.661  2.149  2.048  2.379  5.661  2.149  2.048  2.379  5.661  2.149  2.048  2.379  5.661  2.149  2.048  2.379  5.661 | 19.19%  13.40%  5.80%  4.00%  39.67%  28.08%  7.40%  5.00%  53.63%  33.46%  9.80%  3.80%  62.47%  43.72%  11.20%  5.21%  63.36%  42.28%  13.4%  5.00%  38.91%  26.02%  8.6%  3.00%  15.04%  11.71%  4.42%  2.60% | 33.96%  31.97%  32.65%  31.63%  24.01%  25.53%  26.60%  26.10%  20.57%  20.17%  20.43%  19.11%  16.69%  16.78%  14.78%  16.45%  9.14%  9.46%  8.81%  8.96%  4.69%  4.75%  4.60%  4.79%  2.39%  2.41%  2.32%  2.42% |
| --- | --- | --- | --- |

**Table S2:** Power of simulations for character rate change. Remaining parameters were symmetrical for each simulation (*μ* _0_ **=** 0.03, *μ* _1_ **=** 0.03, λ _0_ **=** 0.1, λ _1_ **=** 0.1). Power is plotted in Fig 1B. The observed percent of terminal taxa with State 0 is indicated by the mean value from 500 simulations.

| Rate of Character Change | 5% Cutoff | Power | Observed State 0 |
| --- | --- | --- | --- |

**Table S3:** Power of asymmetrical extinction rate simulations. Remaining parameters were symmetrical for each simulation (*q*_01_ **=** 0.01, *q*_10_ **=** 0.01, λ _0_ **=** 0.1, λ _1_ **=** 0.1). Power is plotted in Fig 1C. The observed percent of terminal taxa with State 0 is indicated by the mean value from 500 simulations.

| Rate of Extinction | 5% Cutoff | | Power | Observed State 0 |
| --- | --- | --- | --- | --- |
| 2× (*μ*_0_ **=** 0.06, *μ*_1_ **=** 0.03; Ratio 3_1_:1_0_; Expected State 0 = 25%)  500 taxa  300 taxa  100 taxa  50 taxa  3× (*μ*_0_ **=** 0.09, *μ*_1_ **=** 0.03; Ratio 6_1_:1_0_; Expected State 0 = 14%)  500 taxa  300 taxa  100 taxa  50 taxa  4× (*μ*_0_ **=** 0.12, *μ*_1_ **=** 0.03; Ratio 9_1_:1_0_; Expected State 0 = 10%)  500 taxa  300 taxa  100 taxa  50 taxa  5× (*μ*_0_ **=** 0.15, *μ*_1_ **=** 0.03; Ratio 12_1_:1_0_; Expected State 0 = 7.7%)  500 taxa  300 taxa  100 taxa  50 taxa  10× (*μ*_0_ **=** 0.3, *μ*_1_ **=** 0.03; Ratio 27_1_:1_0_; Expected State 0 = 3.5%)  500 taxa  300 taxa  100 taxa  50 taxa | 2.128  2.443  2.869  5.567    2.128  2.443  2.869  5.567  2.128  2.443  2.869  5.567  2.128  2.443  2.869  5.567  2.128  2.443  2.869  5.567 | 11.47%  7.22%  4.60%  4.00%  20.00%  10.02%  5.00%  4.00%  20.78%  6.70%  5.62%  1.20%  15.87  4.47%  2.60%  1.40%  3.71%  2.5%  1.40%  0.20% | | 23.85%  24.22%  23.99%  24.33%  13.21%  13.16%  13.01%  12.69%  9.29%  9.15%  9.05%  8.91%  7.12%  7.17%  7.00%  7.05%  3.40%  3.37%  3.41%  3.04% |

**Table S4:** This table lists statistical power of the BiSSE model for 500 simulations containing 3:1 and 7:1 biases in terminal states for varying tree sizes for likelihood comparisons of power in four versus six parameter models. Power is plotted in Fig 2. Using the stationary frequency formula, in an iterative calculation, we obtained ratios of rates necessary to generate a low bias representative (3_1_:1_0_) and high bias representative (7_1_:1_0_) tip ratios using values symmetrically placed around base rates (λ = 0.1, μ = 0.05, and q = 0.005). For the low bias, rate ratios were 1.1425, 1.3046 and 3.0 for speciation, extinction and character change respectively, yielding simulated rates for speciation λ_0_=0.0936, λ_1_**=** 0.10689, for extinction, μ_0_=0.04378, μ_1_**=** 0.05711, and for character change q_0_= 0.00289, q_1_**=** 0.00866. For the high bias, rate ratios were 1.407, 1.960, and 7.000, yielding simulated rates for speciation λ_0_=0.0843, λ_1_**=**0.1186, for extinction μ_0_=0.0357, μ_1_**=** 0.07, and for character change q_0_=0.00189, q_1_**=** 0.01323. Simulated rates without bias were set to their base rates.

| Speciation | 5% Cutoff (4) | Power (4) | 5% Cutoff (6) | Power (6) |
| --- | --- | --- | --- | --- |

| **Ratio 3_1_:1_0_**  500 taxa  350 taxa  200 taxa  50 taxa  **Ratio 7_1_:1_0_**  500 taxa  350 taxa  200 taxa  50 taxa | 2.149  2.097  2.132  2.371  2.149  2.097  2.132  2.371 | **23.6%**  **17.8%**  **10.2%**  **4.8%**  **70.0%**  **60.4%**  **33.8%**  **9.6%** | 2.019  1.853  1.872  1.786  2.019  1.853  1.872  1.786 | 9.8%  5.6%  5.8%  4.4%  19.2%  20.2%  12%  5.8% |
| --- | --- | --- | --- | --- |

| Character Change |  |  |  |  |
| --- | --- | --- | --- | --- |

| **Ratio 3_1_:1_0_**  500 taxa  350 taxa  200 taxa  50 taxa  **Ratio 7_1_:1_0_**  500 taxa  350 taxa  200 taxa  50 taxa | 2.468  1.898  2.367  2.528  2.468  1.898  2.367  2.528 | **40.2%**  **39.2%**  **16.8%**  3.8%  **65.6%**  **59.6%**  **16.8%**  4.2% | 2.125  1.826  1.888  0.997  2.125  1.826  1.888  0.997 | 29.2%  26.0%  13.2%  **6.2%**  48.0%  41.2%  13.4%  **8.6%** |
| --- | --- | --- | --- | --- |

| Extinction |  |  |  |  |
| --- | --- | --- | --- | --- |

| **Ratio 3_1_:1_0_**  500 taxa  350 taxa  200 taxa  50 taxa  **Ratio 7_1_:1_0_**  500 taxa  350 taxa  200 taxa  50 taxa | 2.305  2.261  2.765  2.472  2.305  2.261  2.765  2.472 | **13.6%**  **11.6%**  **6.2%**  **4.2%**  **43.6%**  **32.6%**  **13.4%**  **6.6%** | 2.048  2.183  1.913  1.608  2.048  2.183  1.913  1.608 | 7.6%  4.6%  4.4%  4.0%  12.8%  6.0%  3.8%  2.4% |
| --- | --- | --- | --- | --- |
