# Supplementary material for: Exploring power and parameter estimation of the BiSSE method for analyzing species diversification
Source: BMC Evol Biol. 2013 Feb 11;13:38. doi: 10.1186/1471-2148-13-38 (PMC3583807; doi:10.1186/1471-2148-13-38)
Supplement: Additional file 2: Figure S1 — Parameter estimations of (a) speciation, (b) character change, and (c) extinction under different tree sizes and degrees of asymmetry in speciation rates with corresponding tip ratios. Point of intersection between red lines represents known values. (PDF 640 kb) [file 1471-2148-13-38-S2.pdf]

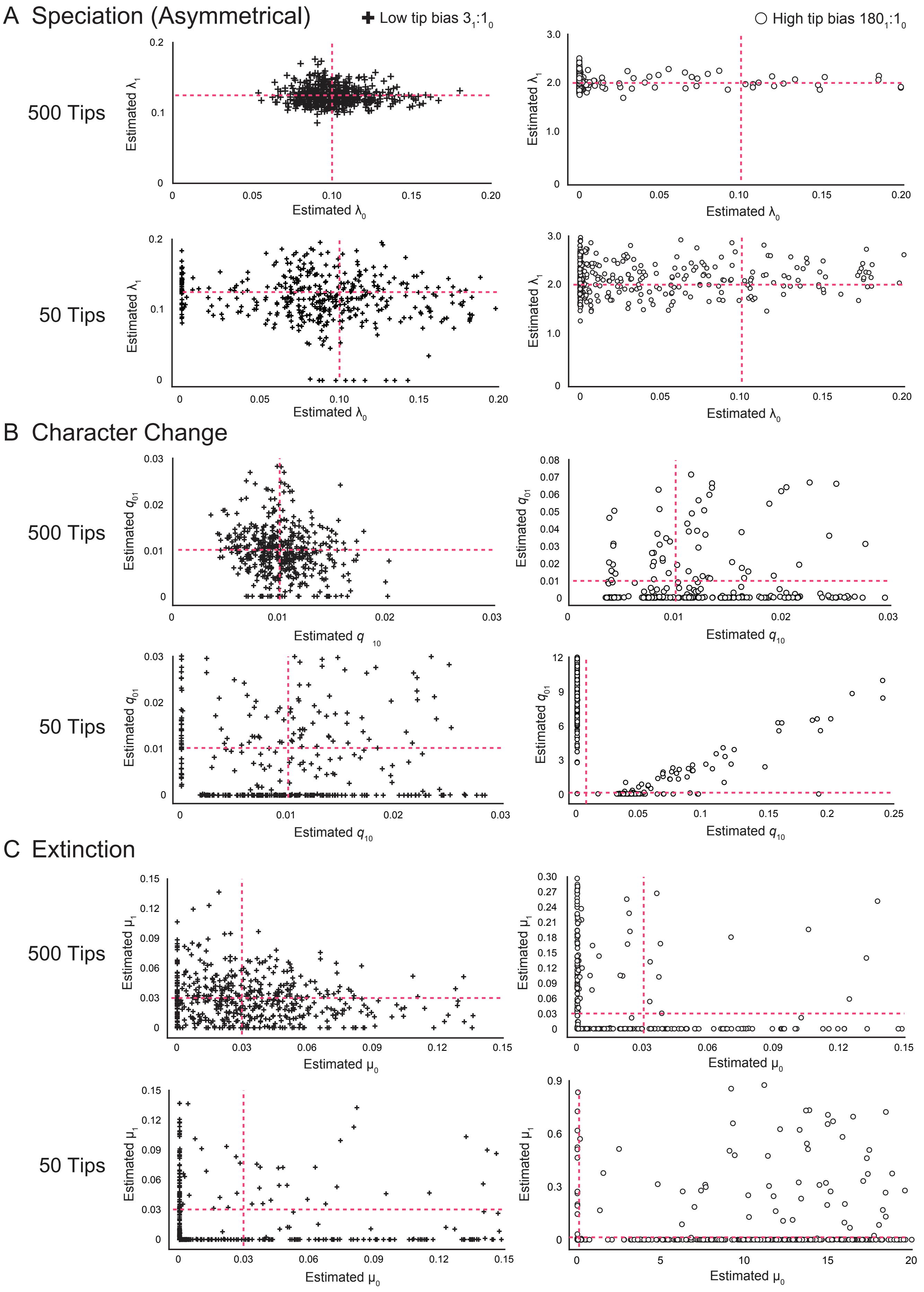

A Speciation (Asymmetrical)
Low tip bias $3_1:1_0$
High tip bias $180_1:1_0$
500 Tips
50 Tips
Estimated $\lambda_1$
Estimated $\lambda_0$
B Character Change
500 Tips
50 Tips
Estimated $q_{01}$
Estimated $q_{10}$
C Extinction
500 Tips
50 Tips
Estimated $\mu_1$
Estimated $\mu_0$
